# Feasibility of combining noninvasive brain stimulation and personalized counseling to increase physical activity

**DOI:** 10.1093/geroni/igab046.3698

**Published:** 2021-12-17

**Authors:** On-Yee (Amy) Lo, Connor Mulvey, Christine Lee, Margaret Gagnon, Lewis Lipsitz, Brad Manor

**Affiliations:** 1 Hebrew SeniorLife/Harvard Medical School, Boston, Massachusetts, United States; 2 Hebrew SeniorLife, Boston, Massachusetts, United States; 3 Hinda and Arthur Marcus Institute for Aging Research, Harvard Medical School, Boston, Massachusetts, United States

## Abstract

Few older adults meet recommended physical activity guidelines. Behavioral interventions may be more effective when combined with other modalities to promote activity. Transcranial direct current stimulation (tDCS) designed to increase the excitability of the left dorsolateral prefrontal cortex (dlPFC) — a brain region subserving motivation and executive function — has the potential to augment behavioral interventions. We designed a randomized, double-blinded trial to examine the feasibility of combining personalized behavioral counseling and tDCS targeting the left dlPFC to improve physical activity and related outcomes in sedentary older adults living within the supportive housing. Participants wore a Fit-Bit throughout the study period. Baseline step counts were determined for two weeks, then participants completed four bi-weekly personalized counseling sessions over eight weeks. They were also randomized to receive 10 sessions of tDCS or sham stimulation over the two weeks after the baseline. Physical, cognitive, and patient-reported outcomes were assessed at baseline, after ten brain stimulation sessions, and after four behavioral sessions. 33 individuals were screened and 16 enrolled (age=80±7, 13 females). 13 participants completed the study, including 100% of study assessments, 99±5% of brain stimulation sessions, and 98±7% of behavioral sessions. Fit-Bit adherence rate was 93±13%. Daily step counts were 3197±1480 at baseline and 4722±2553 over the last two weeks of the intervention. While the study is ongoing and blinded, these preliminary results indicate that it is feasible to conduct a controlled study of tDCS combined with personalized behavioral counseling to increase physical activity in sedentary older adults living within supportive housing.

